# Supplementary figures and images for: Computational Analysis of Therapeutic Neuroadaptation to Chronic Antidepressant in a Model of the Monoaminergic Neurotransmitter and Stress Hormone Systems
Source: Front Pharmacol. 2019 Oct 25;10:1215. doi: 10.3389/fphar.2019.01215 (PMC6823241; doi:10.3389/fphar.2019.01215)

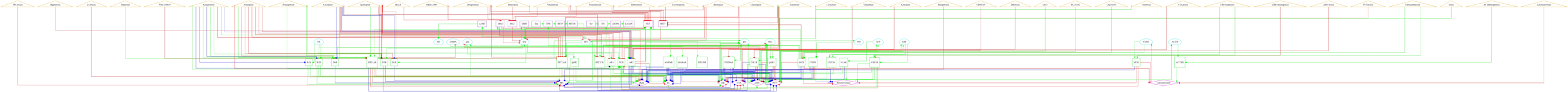

Supplement: Supplementary file 2 [file Image_1.jpeg]
